# Modifiable cardiovascular risk factors among apparently healthy adult Nigerian population - a cross sectional study

**DOI:** 10.1186/1756-0500-3-11

**Published:** 2010-01-20

**Authors:** Mahmoud U Sani, Kolawole W Wahab, Bashir O Yusuf, Maruf Gbadamosi, Omolara V Johnson, Akeem Gbadamosi

**Affiliations:** 1 Department of Medicine, Bayero University, PMB 3011, Kano, Nigeria; 2 Department of Medicine, Aminu Kano Teaching Hospital, Kano, Nigeria; 3 Department of Medicine, University of Ilorin Teaching Hospital, Ilorin, Nigeria; 4 Department of Medicine, Federal Medical Centre, Katsina, Katsina state, Nigeria

## Abstract

**Background:**

Cardiovascular disease (CVD) remains a major cause of morbidity and a leading contributor to mortality worldwide. Over the next 2 decades, it is projected that there will be a rise in CVD mortality rates in the developing countries, linked to demographic changes and progressive urbanization. Nigeria has witnessed tremendous socio-economic changes and rural-urban migration which have led to the emergence of non-communicable diseases. We set out to determine the prevalence of modifiable CVD risk factors among apparently healthy adult Nigerians. This is a descriptive cross-sectional study carried out at Katsina, northwestern Nigeria from March to May 2006. Subjects for the study were recruited consecutively from local residents, hospital staff and relations of in-patients of the Federal Medical Centre, Katsina using convenience sampling. Socio-demographic information, anthropometric measurements and blood pressure were obtained from the subjects in a standardized manner. Venous samples were collected for necessary investigations and analyzed at the hospital central laboratory.

**Findings:**

Three hundred subjects (129 males and 171 females) with a mean age of 37.6 ± 10.6 (range 18-75) years were studied. Prevalence of the modifiable cardiovascular risk factors screened for were as follows: generalized obesity 21.3% (males 10.9%, females 29.2%, p < 0.05), truncal obesity 43.7% (males 12.4%, females 67.3%, p < 0.05), hypertension 25.7% (males 27.9, females 24%, p > 0.05), type 2 diabetes mellitus 5.3% (males 5.4%, females 5.3%, p > 0.05), hypercholesterolaemia 28.3% (males 23.3%, females 32.2%, p < 0.05), elevated LDL-cholesterol 25.7% (males 28%, females 24%, p > 0.05), low HDL-cholesterol 59.3% (males 51.9%, females 65%, p < 0.05), hypertriglyceridaemia 15% (males 16.3%, females 14%, p > 0.05) and metabolic syndrome 22% (males 10.9%, females 30.4%, p < 0.05).

**Conclusions:**

We found high prevalence of CVD risk factors among apparently healthy adult Nigerians. In order to reduce this high prevalence and prevent subsequent cardiovascular events, encouragement of a healthy lifestyle is suggested.

## Introduction

Cardiovascular disease (CVD) remains a major cause of morbidity and a leading contributor to mortality worldwide. The World Health Report 1999 estimates that in 1998, 78% of the burden of non-communicable diseases (NCDs) and 85% of the CV burden arose from the low and middle income countries. The CVD burden afflicts both men and women, with CV deaths accounting for 34% of all deaths in women and 28% in men in 1998[1]. As the epidemics advance, the social gradient also reverses with the poor becoming the most vulnerable victims in both developed and developing countries [2].

The high burdens of CVD in the developing countries are attributable to the increasing incidence of atherosclerotic diseases, perhaps due to urbanization and higher risk factor levels (such as obesity, diabetes, dyslipidemia, hypertension), the relatively early age at which they manifest, the large sizes of the population, and the high proportion of individuals who are young adults or middle-aged in these countries[3]. For example, about half of the deaths attributable to CVD in the developing countries in 1990 occurred below the age of 70 years, in contrast to about a quarter in the developed countries[4]. Such a pattern of premature CVD mortality is likely to haunt the developing countries even more in the future. Between 1990 and 2020, the increase in coronary heart disease (CHD) mortality (120% in women and 137% in men) in the developing countries is expected to be much greater than among developed countries (29% and 48% in women and men respectively)[4].

It is estimated that the elderly population will increase globally (over 80% during the next 25 years), with a large share of this rise in the developing world because of expanding populations. Increased longevity due to improved social and economic conditions associated with lifestyle changes in the direction of a rich diet and sedentary habits, is one of the main contributors to the incremental trend in CVD in the last century[5]. Nigeria has witnessed tremendous socio-economic changes and rural-urban migration which have led to emergence of non-communicable diseases including ischaemic heart disease[6]. The development of CVD is promoted by major risk factors - dyslipidemia, hypertension, diabetes (DM) and smoking. These risk factors are independently associated with CVD risk and are common among adults both in the developed and developing countries. The identification of these major risk factors and the implementation of control strategies (e.g. community education and target of high risk individuals) have contributed to the fall in CVD mortality rates observed in industrialized nations[7]. The non-communicable disease (NCD) survey done in Nigeria about 15 years ago sought to determine the prevalence of major risk factors in the country [8]. With socio-economic changes, rural-urban migration and changes in the definition of the various risk factors, it is likely that the prevalence of these risk factors is increasing. Furthermore, control of infectious, parasitic, and nutritional diseases allows most of the population to reach the ages in which CVD manifests itself. This is the concept of "epidemiologic transition" on which the basis for a prediction of a global CVD epidemic lies[9]. We therefore set out to provide descriptive information regarding the risk factors for CVD in a sample of apparently healthy adult Nigerians, describe how these risk factors relate to one another and also establish a baseline and make appropriate recommendations.

## Methods

This was a descriptive cross-sectional study carried out in Katsina city, in the north western region of Nigeria between March and May, 2006. The city, which has a total population of 459,022 based on 2006 national census figures with a male to female ratio of 1:1.05, is composed majorly of Hausa and Fulani tribes while other Nigerian tribes are found in minority.

Using convenience sampling method, subjects were consecutively recruited after obtaining a written informed consent. The recruited respondents were members of the public, hospital staff and relations of inpatients who were requested to come for a free screening after a health education on the need for prevention of CVD risk factors was given to them by the investigators. Screening was conducted at the Federal Medical Centre, Katsina which is a tertiary hospital that serves as a referral centre for all primary and secondary healthcare facilities in Katsina state.

Subjects were considered apparently healthy if they were asymtomatic, had no physical disability and believed they were in a good state of health. The study protocol was approved by the research and ethics committee of the hospital. In a standardized manner, information was obtained on relevant sociodemographic characteristics like age, sex, current history of alcohol or tobacco use, hypertension and diabetes mellitus with the aid of an interviewer-administered semi-structured questionnaire.

### Anthropometric and blood pressure measurements

All anthropometric measurements were made by 2 trained research assistants. Weight was taken with light clothing on with the aid of a weighing scale with the weights measured to the nearest 100 g, while for measurement of height, a stadiometer was used[10]. Body mass index (BMI) was then calculated from weight (in kilogrammes) divided by a square of the height (in metres). Blood pressure (BP) was measured in the left arm in the sitting position with the aid of a mercury sphygmomanometer using the auscultation method. The systolic blood pressure was recorded at phase I Korotkoff sounds while the diastolic blood pressure was recorded at phase V Korotkoff sounds. All measurements were taken twice and the average of the 2 readings was taken as final.

### Biochemical analysis

After 10-12 hours of overnight fast, venous samples were obtained from the subjects.

The serum was immediately separated by centrifugation, and the concentration of triglycerides (TG), total cholesterol (TC) and its fractions [low-density lipoproteins (LDL-C) and high-density lipoproteins (HDL-C)] were ascertained. Fasting plasma glucose and serum uric acid were also measured.

### Definition of Risk Factors

Hypertension was taken as a positive history, use of antihypertensive drugs or persistently elevated BP (>140/90 mmHg) while DM was regarded as a positive history, use of hypoglycaemic agents or a fasting plasma glucose > 126 mg/dl (7.0 mmol/L). Total cholesterol was considered to be high if it was >200 mg/dl. LDLc was considered to be raised if it was >130 mg/dl, triglycerides was considered to be high if it was > 150 mg/dl and HDLc was considered to be low if it was < 40 mg/dl and <50 mg/dl in men and women respectively. Obesity was defined with the BMI using the WHO classification[11]. Metabolic syndrome was defined using the national cholesterol education programme (NCEP) adult treatment panel (ATP) III criteria[12]. According to this criteria a subject has metabolic syndrome if he or she has three or more of the following: waist circumference >102 cm in men and >88 cm in women; triglyceride levels ≥ 150 mg/dl; HDL cholesterol concentration <40 mg/dl in men and <50 mg/dl in women; blood pressure ≥ 130/85 mmHg; and fasting plasma glucose value ≥110 mg/dl. Atherogenic index (AI) was calculated as the ratio of HDLc to TC. High risk was defined as HDLc/TC < 0.18, while average and low risk was 0.18 to 0.40 and >0.40 respectively, according to the European Atherosclerosis society guidelines[13].

### Statistical Analysis

Data analysis was done using the *Statistical Package for the Social Sciences *(SPSS), version 13.0. Analysis of continuous variables was carried out using the procedures of descriptive statistics and later, to identify any differences we used Student's "*t" *test. Categorical variables were analyzed using contingency tables involving chi-square (*X*^2^) tests to identify statistical differences between the genders. In order to determine the correlation of each of the clinical and laboratory characteristics of the respondents with atherogenic index, Pearson's correlation coefficient was done. Statistical significance was set at the conventional *p *< 0.05.

## Results

**Figure 1:**
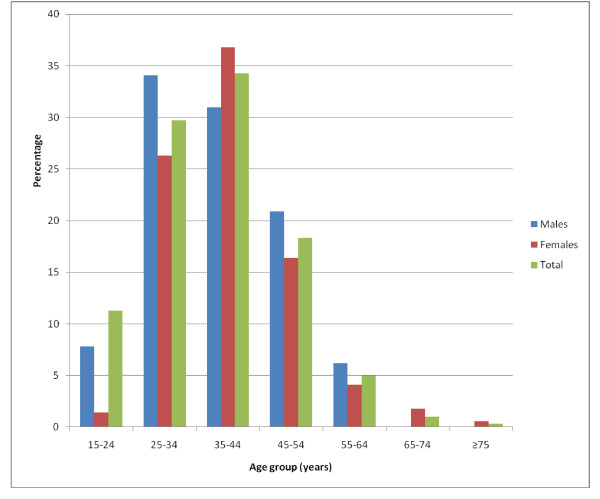
**Age and sex distribution of respondents**.

A total of 321 subjects turned out for the study of whom 300 had complete data for analysis. There were 129 males and 171 females (ratio 1:1.3); and their ages ranged from 18-75 (mean 37.6 ± 10.6) years. Figure 1 shows the age and sex distribution of the study subjects. Most (34.3%) of the study subjects were in the 35-44 year age group. There were more females than males across all age groups. The males were aged 18-58 years (mean 38.0 ± 9.7) while females were aged 18-75 (mean 37.2 ± 11.2), p > 0.05.

There were 48 known hypertensives subjects of whom 40 were on treatment. Nineteen (47.5%) of those on treatment had their BP under control. Twenty nine (9.7%) of the subjects were newly discovered to have hypertension. This together with the known hypertensives makes the hypertension prevalence among the study subjects to be 25.7% (77/300). Ten of the subjects were cases of type 2 DM, out of whom 8 were on treatment and 5 had good glycaemic control. Six subjects were newly found have type 2 DM altogether making type 2 DM to be present in 16 (5.3%).

Sixty four (21.3%) of the subjects were obese (BMI > 30 kg/m^2^) while 96 (32%) were overweight (BMI 25-29.9 kg/m^2^). Across all grades of obesity and the overweight class, females were significantly more affected than males (p < 0.05). Sixteen (12.4%) males and 115 (64.2%) females had increased waist circumference more than 102 cm and 88 cm respectively. Cigarette smoking was found in 14 (4.7%) of whom 11 (79%) were males. Alcohol consumption was generally low and was present in 17 (5.7%) subjects, 14 (82%) of whom are males. The means of clinical and laboratory characteristics of the subjects are shown in Table 1.

**Table 1:** Means of Clinical and Laboratory Characteristics of the subjects

Variable	Overall (n = 300)	Male (n = 129)	Female (n = 171)	p value
Age, years	37.6 ± 10.6	38.0 ± 9.7	37.2 ± 11.2	0.54
BMI, kg/m^2^	26.0 ± 5.8	24.1 ± 4.0	27.5 ± 6.5	< 0.001
WC, cm	91.1 ± 14.5	87.6 ± 10.9	93.7 ± 16.2	< 0.001
Systolic BP, mmHg	120.4 ± 21.1	117.9 ± 18.9	122.3 ± 22.4	0.07
Diastolic BP, mmHg	78.9 ± 13.5	77.4 ± 11.8	80.1 ± 14.6	0.10
FPG, mg/dl	82.9 ± 20.9	82.9 ± 15.4	82.9 ± 24.2	0.99
TC, mg/dl	178.2 ± 38.0	172.8 ± 35.6	182.3 ± 39.4	0.03
HDL cholesterol, mg/dl	45.3 ± 21.7	40.1 ± 14.6	49.2 ± 25.1	< 0.001
LDL cholesterol, mg/dl	106.8 ± 38.9	105.7 ± 38.1	107.7 ± 39.5	0.66
Triglyceride, mg/dl	101.3 ± 49.1	104.4 ± 46.2	99.1 ± 51.2	0.36
HDLc/TC	0.25 ± 0.12	0.24 ± 0.11	0.27 ± 0.13	0.02

Values are Mean ± SD. BMI = body mass index, WC = waist circumference, BP = blood pressure, FPG = fasting plasma glucose, TC = fasting total cholesterol, HDL = high density lipoprotein, LDL = low density lipoprotein

Table 2 shows the overall and gender frequencies of the modifiable cardiovascular risk factors. Eighty five (28.3%) had raised total cholesterol, 77 (25.7%) had raised LDLc and 67 (52%) male and 111 (65%) females had low HDLc. Trigycerides was high in 45 (15%) of the subjects. Thirty eight (29.5%) males and 39 (22.8%) females had CHD risk ratio in the high risk category (HDLc/TC < 0.18). Metabolic syndrome was present in 66 (22%) of the subjects, 52 (30.4%) of whom were females (p < 0.001).

**Table 2:** Frequency of cardiovascular disease risk factors, metabolic syndrome and artherogenic index.

CV risk factor	Overall, n (%)	Male, n (%)	Female, n (%)	p value
Hypertension	77(25.7)	36(27.9)	41(24.0)	0.23
DM	16 (5.3)	7(5.4)	9(5.3)	0.56
BMI >30 kg/m^2^	64 (21.3)	14 (10.9)	50(29.2)	< 0.001
Truncal obesity	131(43.7)	16(12.4)	115(67.3)	< 0.001
Hypercholesterolaemia	85(28.3)	30(23.3)	55(32.2)	< 0.05
Raised LDLc	77(25.7)	36(28.0)	41(24.0)	0.59
Low HDLc	178(59.3)	67 (51.9)	111(65.0)	< 0.001
Hypertriglyceridaemia	45(15.0)	21(16.3)	24 (14.0)	0.59
Cigarette smoking	14(4.7)	11(8.5)	3 (1.8)	< 0.001
Metabolic syndrome	66(22.0)	14(10.9)	52(30.4)	< 0.001
HDLc/TC (< 0.18)	77(25.7)	38(29.5)	39(22.8)	0.86

DM; Diabetes Mellitus, BMI; body mass index, LDLc; low density lipoprotein cholesterol, HDLc; high density lipoprotein cholesterol, TC; total cholesterol, HDLc/TC; atherogenic index.

Table 3 shows that CHD risk ratio correlated negatively with TC (r = -0.3, p < 0.05), LDLc (r = -0.5, p < 0.05) and WC (r = -0.2, p < 0.05) and correlated positively with HDLc (r = 0.9, p < 0.05).

**Table 3:** Clinical and laboratory characteristics with significant correlation with artherogenic index

Cardiovascular risk factor	Pearson's correlation coefficient (r)	p value
Waist circumference	-0.20	0.001
Weight	-0.16	0.005
Total cholesterol	-0.30	< 0.001
HDLc	0.87	< 0.001
LDLc	-0.50	< 0.001
Metabolic syndrome	-0.20	0.001

Note: Artherogenic index = HDLc/TC

## Discussion

Developing countries face the double menace of prevalent infectious diseases and increasing CVD with projected epidemic proportions in the near future. CVD has become the number-one cause of death in the developing world, causing twice as many deaths as HIV, malaria, and tuberculosis combined[14]. This epidemic has the potential to place a large social and economic burden on developing countries, where CVD tends to strike those in their prime of life[15]. In developing countries, the increase in CVD burden is largely the result of an increase in the prevalence of risk factors and a relative lack of access to various interventions used to achieve successes in developed countries.

Many earlier studies in Nigeria sought to describe prevalence of risk factors for CVD in specific populations like those with hypertension, diabetes and the aged [16-18]. The present study, in addition to providing descriptive information on CVD risk factors in a sample of apparently healthy adult Nigerians, has also tried to establish to what extent these risk factors relate to one another.

The NCD survey of CV risk factors in Nigeria conducted 15 years earlier reported the age-adjusted prevalence of hypertension to be 11.2%, using a qualifying BP level of 160/95 mmHg[8]. Using the same qualifying BP level, other workers found the prevalence of hypertension to be 12.4% in south western Nigeria [19]. Extrapolations from the NCD data and from other studies suggest an urban prevalence rate of around 20% using the current cut-off point of 140/90 mmHg [20,21]. In a survey of cardiovascular risk factors in middle aged Nigerians aged 50-54 years, hypertension was found in 25% of males and 16.4% of females [18]. The prevalence of hypertension found in this study is comparable to that found by these other workers in the recent past. This shows that the prevalence of hypertension in Nigeria has increased when compared with the findings from the NCD survey 15 years ago.

The prevalence of type 2 DM was 5.3%, which is higher than the 2.2%-2.8% found by other workers in the country[8,18,22]. Rotimi and colleagues reported the age adjusted prevalence of type 2 DM to be 1%, 12% and 13% among Nigerians, Jamaicans and US blacks respectively[23]. There have been comments and projections about the rising incidence of diabetes worldwide[24]. It is important to note that in the present study, there is a positive correlation between the measures of obesity (BMI and waist circumference) and fasting plasma glucose. It is possible that the high prevalence of DM in this study is a reflection of high prevalence of both generalized and truncal obesities since the latter is an indirect measure of insulin resistance which is the hallmark of type 2 DM. However, although BMI and waist circumference are significantly higher in our female subjects, the reason for lack of difference in the gender prevalence of DM is not apparent.

As compared to men, women had higher BMI (27.5 versus 24.1 kg/m^2^, p < 0.05). This agrees with findings of other studies in the Nigeria[18] and elsewhere in Africa[25]. Waist circumference (WC) is an index of truncal obesity and when increased is unfavourably associated with cardiovascular disease risk. The WC showed women were more obese than men in agreement with other studies[18,25]. The high proportion of obese subjects especially among women is likely due to the fact that obesity is still "fashionable" in Nigeria, especially among women as it is thought to depict affluence[26]. In addition, traditional African culture does not encourage physical exercise especially among women. Most of the subjects had sedentary lifestyles which encourage weight gain.

There is a high prevalence of dyslipidaemia in our subjects with the most common in both sexes being low HDL-cholesterol. In agreement with the results of other studies[18,25], the females presented a higher proportion of those with significantly higher concentrations of TC and reduced levels of HDLc fraction.

Metabolic syndrome was present in 22% which is comparable to the 21.8% reported in the USA[27]; however, this prevalence is lower compared to the findings of Ker et al[28] in South Africa among corporate executives using the same criteria like we used in our study. The difference could possibly be as a result of the fact that their study was among corporate executives who were already at increased risk for metabolic syndrome because of the sedentary nature of their job. The higher frequency of metabolic syndrome among our female subjects is also comparable to the findings of Gupta et al[29] (47.8% vs 36.2%) and Azizi et al[30] (42% vs 24%).

Almost 1 out of every 4 individuals screened had atherogenic index below 0.18 which implies that they are at high risk of CHD and this did not show any gender disparity. This finding is of great concern because CHD was thought to be rare in Nigeria. The high prevalence of those with atherogenic index in the high risk category is expected because of the high prevalence of dyslipidaemia in the study subjects. Nigeria is witnessing both epidemiologic and demographic transitions and the high rate of CVD risk factors in our subjects should serve as an eye opener to the health workers and policy makers on the need for a strategic plan to prevent this from getting to an epidemic dimension.

We acknowledge that our study is limited in a number of ways. Firstly the sample size is small for this kind of study and this could have increased the probability of type 2 error. Secondly, the non-probability sampling technique used could have possibly increased the risk of bias. As such, the results cannot be generalized to the entire population. We however feel that because our respondents were recruited consecutively without any selection, the chances of bias was reduced. Additionally, as we did not screen for other established CVD risk factors like microalbuminuria, homocysteinaemia and C reactive protein, the relative contributions of each of these to the burden of cardiovascular disease in our environment could not be determined. The findings of this study can serve as a template for a proper community-based study on the same subject in the country, especially in view of the high prevalence of the CVD risk factors screened for.

## Conclusions and Recommendations

This study has shown that there is a high prevalence of cardiovascular risk factors among our study population, with females having significantly higher prevalence rates compared to males. Also, as a result of this high prevalence of CVD risk factors, almost 1 out of every 4 apparently healthy individuals in Nigeria could be at high risk of CHD. It is therefore important for us to develop a proactive approach to managing the looming epidemic of cardiovascular diseases. We also need to promote lifestyle-related disease control and prevention programmes which include intake of healthy diet, regular aerobic exercises and decreased tobacco and alcohol use.

## Competing interests

We have no conflict of interest to declare. This research received no specific grant from any funding agency in the public, commercial or not-for-profit sectors. We also have no financial disclosure.

## Authors' contributions

MUS and KWW conceived and designed the study. BOY, MG, OVJ and AG were involved in initial literature search, all authors were involved in data collection. MUS drafted the initial manuscript while KWW read the initial manuscript for major intellectual input. All authors read and approved the final draft.
